# A clinical future for the myocardial phosphocreatine/adenosine triphosphate ratio?

**DOI:** 10.1016/j.jocmr.2026.102694

**Published:** 2026-01-18

**Authors:** Adrianus J. Bakermans

**Affiliations:** Department of Radiology and Nuclear Medicine, Amsterdam University Medical Center, University of Amsterdam, Amsterdam, the Netherlands

Adequate maintenance of myocardial energy homeostasis is crucial for the heart’s capacity to perform the mechanical work that powers the blood circulation. At physiological concentrations, hydrolysis of adenosine triphosphate (ATP) into adenosine diphosphate and inorganic phosphate (P_i_) provides the chemical energy to support myocardial contraction and relaxation. Catalyzed by the creatine kinase enzyme, dephosphorylation of phosphocreatine (PCr) provides rapid buffering of the ATP pool. Vice versa, creatine kinase catalyzes the replenishment of the PCr pool from ATP produced in mitochondria. It is clear that the ability to detect alterations in the steady-state tissue concentrations of these metabolites would provide important insight into myocardial energy homeostasis, as such offering great potential for early monitoring of disease progression and therapeutic response. The seminal work by Bottomley in the 1980s [1] started to unlock this potential by presenting a noninvasive approach to acquire localized phosphorus-31 magnetic resonance (^31^P-MR) spectra from the in vivo human heart. To date, ^31^P-MR spectroscopy (^31^P-MRS) remains the only quantitative and nondisruptive modality for evaluating in vivo tissue high-energy phosphate metabolism. While magnetic resonance imaging has evolved over these decades into the gold standard for quantitative imaging of myocardial anatomy and function, ^31^P-MRS has not. Despite the many studies that demonstrated its ability to detect impaired myocardial energy homeostasis, and despite many ongoing technological advances, ^31^P-MRS of the human heart remains a niche application that has value in research [2], but that has not matured into clinical applications for disease diagnosis and monitoring.

To provide an evidence base for the potential integration of ^31^P-MRS into the clinic, Peng et al. [3] recently conducted a literature meta-analysis deriving an association between the ratio of myocardial PCr and ATP concentrations measured with ^31^P-MRS and the presence and severity of heart failure. The PCr/ATP concentration ratio, which reflects the in vivo myocardial energy status, is somewhere around 1.7 for the healthy human heart [4] and is under tight homeostatic control, but is not sensitive to a simultaneous reduction of both PCr and ATP concentrations. Based on 26 identified studies that included both patients with heart failure (totaling *n* = 654) as well as a control group (*n* = 521), this meta-analysis now shows that a 1-point decrease of the PCr/ATP ratio comes with an odds ratio of 7.6 (95% confidence interval, 4.9-11.8) for heart failure. The work by Peng et al. reassuringly provides strong confirmatory evidence that a consistent association exists between a reduced myocardial energy status and the presence of heart failure, despite physiological and methodological heterogeneity among studies. Based on this association, these authors propose that myocardial PCr/ATP is a good candidate as a clinical biomarker of (early) impairment of myocardial energy homeostasis, while also advocating for the standardization of acquisition and processing protocols for estimating myocardial PCr/ATP with ^31^P-MRS. Indeed, the methodological variability in reported ^31^P-MRS approaches includes differences in magnetic field strength, transmit and receive coil setups, sequences for localized signal acquisition, corrections for contamination with ATP signal from blood, corrections for partial saturation due to differences in the T_1_ relaxation time constants for PCr and ATP, and differences in signal processing and quantification. A subgroup analysis revealed that effect-size heterogeneity was substantially lower for studies conducted at magnetic field strengths >1.5T (*n* = 12; i^2^ = 18.8%) than for those conducted at 1.5T (*n* = 14; i^2^ = 72.8%). It is tempting to interpret this observation as an indication that a higher magnetic field strength will yield more consistent results with ^31^P-MRS. We note, however, that of these 12 studies at >1.5T, nine were conducted by the same laboratory, and that likely their methodological choices, in general rather than the higher magnetic field strength per se, contributed to the reduced heterogeneity among their studies. As such, Peng et al.’s call for ^31^P-MRS protocol standardization is welcomed, and it is encouraging that this team of vendor-employed authors recognizes the important role that clinical MR vendors have in achieving ^31^P-MRS measurement reliability.

Yet, for ^31^P-MRS-based estimates of the myocardial PCr/ATP ratio to become of any clinical merit, measurements need to be precise and accurate enough to allow detection of a small change in the PCr/ATP ratio in a single subject, or quantification of a small difference against an established reference value. A 1-point decrease of myocardial PCr/ATP would probably be lethal and therefore does not reflect realistic conditions. Recognizing this, Peng et al. rescaled the effect size to show that heart failure odds increase by 23% for a 0.1-point decrease of the myocardial PCr/ATP ratio (odds ratio, 1.23; 95% confidence interval, 1.17-1.28). Unfortunately, current state-of-the-art ^31^P-MRS of the human heart is not nearly sensitive enough to detect such small differences on an individual-patient basis. To achieve this level of reliability, improvements in measurement precision (i.e., repeatability) are required through methodological and technological advancements. Furthermore, standardization of adequately established protocols can help reduce heterogeneity and improve reproducibility among different laboratories. At present, ^31^P-MRS of the heart often requires dedicated (sometimes custom-built) hardware, sequences, and processing pipelines that are constrained by and tailored to laboratory-specific facilities and expertise. These aspects complicate any protocol standardization and may put any heterogeneous results under increased scrutiny [5]. Still, many other factors that influence a quantitative estimate of the myocardial PCr/ATP ratio are difficult if not impossible to control for and could hamper a practical clinical implementation. Unknown blood signal contamination raises the question whether we will need concomitant blood sampling to determine blood ATP content, similar to determining hematocrit for calculating myocardial extracellular volume fractions. Will we need subject-specific estimates of metabolite T_1_ relaxation time constants to correct for partial saturation? And will the ^31^P-MRS procedure become fast and comfortable enough to allow its routine clinical use? Moreover, validation of the technique against biochemical assays to establish its accuracy is essentially impossible, complicating the determination of ground-truth reference values for the human myocardial PCr/ATP concentration ratio. For their meta-analysis, Peng et al. mitigated this lack of normal values by only considering effect sizes in studies that reported both on patients with heart failure as well as on healthy control subjects.

With recent and ongoing impressive improvements of ^31^P-MRS methodology, other readouts may come within reach that are more sensitive to impairment of myocardial energy homeostasis, and that potentially offer more diagnostic value than the PCr/ATP ratio, such as estimation of the ATP synthesis rate through creatine kinase [6], detection of the myocardial P_i_ pool [7], [8], or absolute quantification of metabolite concentrations, e.g., for calculating the Gibbs free energy change (ΔG) of ATP hydrolysis [2]. It remains to be seen whether the long-recognized potential for ^31^P-MRS measurement of myocardial energy homeostasis will ever be fully unlocked. The key lies with our community of researchers, physicists, engineers, clinicians, and vendors to keep improving and facilitating ^31^P-MRS methodology and to showcase relevant applications to integrate the unique possibilities of this modality into the clinic.

## Funding

The work on ^31^P-MR spectroscopy in Dr. Bakermans’ lab is supported in part by the United States 10.13039/100000002National Institutes of Health (NIH; subaward to R01 HL173346). The content is solely the responsibility of the author and does not necessarily represent the official views of the NIH.

## Declaration of competing interests

The author declares the following financial interests/personal relationships which may be considered as potential competing interests: Adrianus J. Bakermans reports financial support was provided by the United States National Institutes of Health. Given his role as Editorial Board Member, Dr. Bakermans had no involvement in the peer review of this article and had no access to information regarding its peer review. Full responsibility for the editorial process for this article was delegated to another journal editor.
